# Large Language Model–Based Clinical Decision Support for Antibiotic Selection and Dose Recommendation in Hospitalized Patients With Pneumonia: Multicenter Retrospective Study

**DOI:** 10.2196/98207

**Published:** 2026-08-04

**Authors:** Yang Zhang, Li Li, Chunting Tan, Mengyuan Ji, Xican Tian, Xiangdong Mu, Jun Li, Yu Gu, Honglei Liu

**Affiliations:** 1 School of Biomedical Engineering, Capital Medical University, No. 10 Xitoutiao, You’anmenwai, Fengtai District, Beijing, 100069, China, 86 010-83911542; 2 Beijing Key Laboratory of Clinical Engineering Solutions for Mental Health, Capital Medical University, Beijing, China; 3 Department of Respiratory and Critical Care Medicine, School of Clinical Medicine, Tsinghua Medicine, Beijing Tsinghua Changgung Hospital, Tsinghua University, Beijing, China; 4 Department of Respiratory Medicine, Beijing Friendship Hospital, Capital Medical University, Beijing, China

**Keywords:** large language models, retrieval-augmented generation, pneumonia, medication recommendation, knowledge graph

## Abstract

**Background:**

Pneumonia is a common infectious disease, and antibiotic treatment in hospitalized patients must balance efficacy, safety, and resistance risk. However, antibiotic selection and dose adjustment still rely heavily on clinician experience. Although large language models (LLMs) are promising for clinical reasoning, their direct use for antibiotic selection and dose recommendation is limited by hallucinations and weak adherence to clinical constraints.

**Objective:**

This study aimed to develop and externally validate a constrained LLM-based clinical decision support pipeline for antibiotic selection and dose recommendation in hospitalized patients with pneumonia.

**Methods:**

We conducted a multicenter retrospective study using electronic health record narratives, antibiotic orders, and laboratory indicators of hepatic and renal function from 331 hospitalized patients with pneumonia from 2 hospitals in China. The development cohort included 233 patients, and the external validation cohort included 98 patients. The pipeline integrated dual-branch retrieval (similar-case vector retrieval plus guideline-based knowledge graph retrieval), clinician-defined rule constraints, and hybrid-context reasoning. DeepSeek-V3, GLM-4.6, and GPT-4o were evaluated using *F*_1_-score and Jaccard accuracy.

**Results:**

On the internal test set, the full pipeline using DeepSeek-V3 achieved the best performance, with an *F*_1_-score of 0.8110 (95% CI 0.7371-0.8762) and Jaccard accuracy of 0.7624 (95% CI 0.6810-0.8386) for antibiotic selection and an *F*_1_-score of 0.7538 (95% CI 0.6671-0.8329) and Jaccard accuracy of 0.7076 (95% CI 0.6145-0.7938) for joint antibiotic selection plus dosing recommendation. On the external validation set, performance remained high, with an *F*_1_-score of 0.8605 (95% CI 0.7891-0.9252) and Jaccard accuracy of 0.8571 (95% CI 0.7857-0.9184) for antibiotic selection, and an *F*_1_-score of 0.8503 (95% CI 0.7789-0.9150) and Jaccard accuracy of 0.8469 (95% CI 0.7755-0.9133) for antibiotic selection plus dosing recommendation. The system also provided traceable evidence and rule trigger information to support clinician review.

**Conclusions:**

A constrained, retrieval-augmented LLM pipeline improved the consistency and interpretability of antibiotic selection and dose recommendation for hospitalized patients with pneumonia and provided preliminary evidence of cross-site generalizability.

## Introduction

Pneumonia is a major infectious disease worldwide and remains a leading cause of morbidity and mortality. Accordingly, standardized diagnosis, treatment, and antimicrobial management are essential components of modern health care. In China, the burden of community-acquired and hospital-acquired pneumonia remains substantial, driven in part by rapid population aging and increasing numbers of immunosuppressed patients and patients with multimorbidity [1]. At the same time, the evolution of bacterial resistance continues to outpace the development and clinical availability of new antimicrobials, making inappropriate antibiotic use a major global public health challenge [2]. In real-world practice, inappropriate antimicrobial prescribing remains common and may compromise treatment efficacy, increase the risk of adverse drug events, accelerate the spread of antimicrobial resistance, and raise health care costs [3]. For hospitalized patients with pneumonia, antibiotic selection often requires early empirical treatment before definitive microbiological results become available. At the same time, patient-specific factors such as hepatic or renal impairment, older age, and multimorbidity can substantially influence antibiotic selection and dose recommendation. These challenges highlight the need for clinical decision support (CDS) tools that can assist individualized antimicrobial therapy while remaining aligned with guideline constraints.

Before the advent of large language models (LLMs), biomedical informatics had long explored CDS approaches. Early rule-based and knowledge-based expert systems [4,5] encoded medical knowledge as “if-then” rules with strong interpretability but relied heavily on manual maintenance and were difficult to update in response to evolving guidelines, emerging evidence, and complex clinical contexts. Subsequently, traditional natural language processing and machine learning methods were applied to tasks such as clinical text structuring, risk prediction, and adverse event detection [6-8]. Deep learning models, including recurrent neural networks and bidirectional encoder representations from transformers, further improved clinical text representations [9-13]. However, prediction or classification remained the dominant paradigm at this stage, often without providing an auditable chain of clinical reasoning [10,14,15]. In the area of medication recommendation and individualized prescribing, previous studies have mainly focused on medication information extraction, prescription review alerts, or medication prediction [6,16,17], with limited ability to jointly incorporate guideline constraints, patient-specific variation, and cross-modal clinical evidence. In addition, limited interpretability and traceability have remained major barriers to clinical adoption in high-risk medication decisions.

In recent years, advances in LLMs for medical context understanding and text generation have expanded biomedical informatics from “structuring and prediction” toward “clinical text reasoning and explainable generation” [18,19]. These models have shown promise in tasks such as medical record information extraction and clinical question answering [20-22]. For medication recommendation, LLMs can integrate illness descriptions, prior medications, and test results to generate candidate regimens and rationales [23-26]. With retrieval-augmented generation, external sources such as guideline statements, drug labels, and local clinical pathways can be incorporated into the reasoning process, thereby improving knowledge coverage and output consistency [27-29]. However, in high-risk settings such as antibiotic selection and dose recommendation, important safety challenges remain. Adherence to fine-grained constraints, including dose limits, contraindications, and hepatic or renal dose adjustment, is often unstable [26]. Model conclusions may also vary according to input phrasing and context organization [23], and hallucinations may produce potentially unsafe recommendations [30]. To support safe antimicrobial decision-making, LLM outputs should be grounded in traceable evidence, guided by explicit clinical constraints, and accompanied by reasoning that can be reviewed and verified by clinicians. These limitations highlight the need for constrained, traceable, and auditable LLM-based frameworks that can systematically incorporate external evidence and explicit clinical rules into the reasoning process.

Against this background, we developed and validated an LLM-based CDS pipeline for antibiotic selection and dose recommendation in hospitalized patients with pneumonia. Designed as an assistive tool for antimicrobial stewardship and medication review rather than a replacement for clinician judgment, the proposed framework is conceptually aligned with the information-gathering process of infectious disease consultation, in which clinicians integrate patient characteristics, laboratory findings, prior experience, and guideline recommendations to support empirical antimicrobial decisions. To support this process, the framework combines two key strategies to improve the safety and consistency of LLM-based recommendations: (1) a dual-branch retrieval mechanism that integrates similar-case vector retrieval with guideline-based knowledge graph retrieval to connect patient-specific information with external evidence and (2) clinician-defined rule constraints that explicitly encode dose adjustment, contraindications, and hepatic or renal function considerations. We further evaluated this framework on multicenter real-world data through internal testing and external validation, with comparisons across multiple LLMs (DeepSeek-V3, GLM-4.6 [Z.ai], and GPT-4o [OpenAI]). By generating recommendations together with traceable evidence and rule trigger information, the proposed system aimed to support rapid clinician verification and final decision-making while improving the interpretability, robustness, and clinical applicability of LLM-assisted antimicrobial management.

## Methods

### Study Design and Datasets

We conducted a multicenter retrospective study using electronic health record narratives, antibiotic orders (including prescription elements such as dose and route), and biochemical laboratory reports from hospitalized patients with pneumonia at 2 hospitals in China. Beijing Tsinghua Changgung Hospital served as the development cohort (n=233; January 2020-January 2024) and was split into training and internal test sets using a 7:3 ratio. Beijing Friendship Hospital served as the external validation cohort (n=98; April 2019-September 2019).

Inclusion criteria were as follows: (1) an admission diagnosis of community-acquired or hospital-acquired pneumonia, (2) complete clinical records, and (3) at least one antibiotic order during hospitalization. The exclusion criterion was confirmed COVID-19 pneumonia.

We constructed the dataset for model input and evaluation using three data domains: (1) patient context, including admission narratives such as the chief concern, history of present illness, physical examination, and initial diagnosis; (2) medication orders, including antibiotic prescriptions and related elements such as drug name, dose, route, and dosing frequency where available; and (3) laboratory indicators, including 7 routinely used hepatic and renal function indicators closely related to antimicrobial safety (Table S1 in Multimedia Appendix 1), which were used to support dose recommendation and safety assessment.

For evaluation, raw antibiotic orders were not directly used as the gold standard but served as source information for reference label construction. Two experienced respiratory physicians reviewed the admission records and laboratory results to determine the final reference regimens. Therefore, model performance was evaluated against expert-adjudicated reference regimens rather than unreviewed raw electronic health record antibiotic orders.

### Model

#### Overview

**Figure 1.**
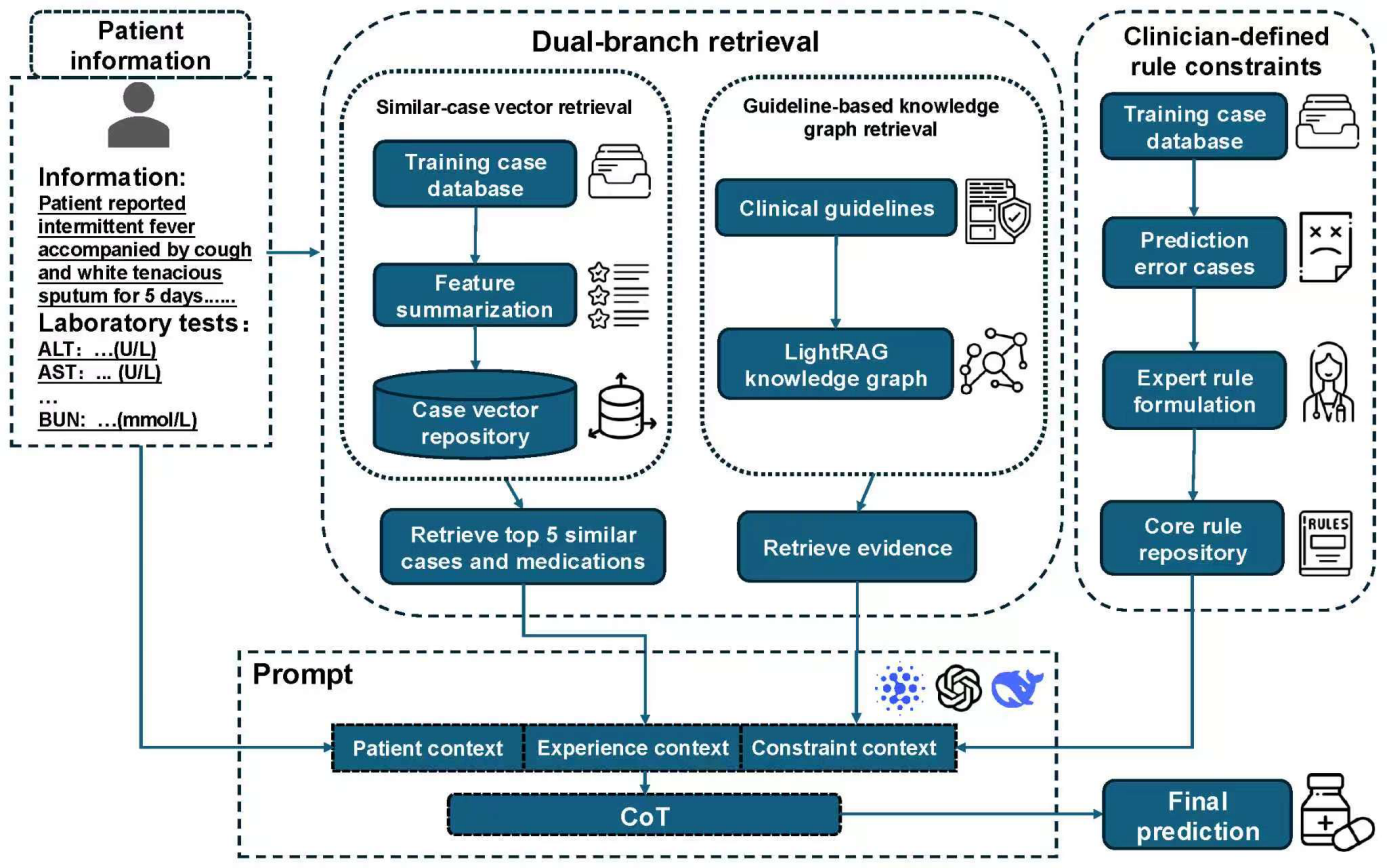
Systematic architecture of the proposed retrieval-augmented medication decision pipeline. ALT: alanine aminotransferase; AST: aspartate aminotransferase; BUN: blood urea nitrogen; CoT: chain of thought.

This study aimed to develop and validate a retrieval-augmented medication decision pipeline for antibiotic selection and dose recommendation. The pipeline takes admission notes and 7 hepatic and renal function indicators as inputs and consists of three core components: (1) a dual-branch retrieval framework, which integrates similar-case vector retrieval with guideline-based knowledge graph retrieval; (2) clinician-defined rule constraints, in which clinician-curated rules are injected as explicit constraints; and (3) a hybrid-context reasoning module, which integrates patient-specific information, retrieved evidence, and rule constraints to generate antibiotic and dose recommendations with traceable supporting evidence for clinician review. The systematic architecture and workflow of the framework are illustrated in Figure 1.

#### Dual-Branch Retrieval Framework

##### Similar-Case Vector Retrieval

To enable robust retrieval from noisy clinical narratives, we adopted a summarize-embed-retrieve workflow. For each historical case, an LLM first generated a structured clinical summary from the admission note and laboratory report covering (1) baseline characteristics, such as demographics, symptoms, and vital signs; (2) examinations and tests, such as imaging findings, infection-related markers, and hepatic and renal function, with laboratory abnormalities discretized into 4 levels (normal, mild, moderate, and severe); and (3) medical history, such as comorbidities, allergies, and prior medications. These summaries were then encoded into dense vectors using the pretrained embedding model bge-m3 to construct a case memory. For a new patient, cosine similarity was calculated against the case memory, and the top 5 most similar cases together with their antibiotic regimens were retrieved as experience-based context for downstream reasoning. The top-5 setting was chosen empirically to balance similar-case coverage with contextual noise and prompt length constraints. The similar-case retrieval configuration and the prompt template used for similar-case summarization are provided in Multimedia Appendix 1.

##### Guideline-Based Knowledge Graph Retrieval

To incorporate evidence-based knowledge and explicit clinical constraints, we used authoritative clinical guidelines, including the Chinese Guidelines for the Diagnosis and Treatment of Community-Acquired Pneumonia in Adults (2016 edition) [31]. Using the LightRAG framework, we performed structured extraction of guideline text to construct a decision-oriented knowledge graph containing 1637 entities and 2077 semantic relations. The detailed parameters are provided in Multimedia Appendix 1.

The workflow included 3 steps. First, key medical concepts were extracted and normalized, including clinical symptoms, disease classification and severity, pathogen risk, drugs and administration routes, hepatic and renal function status, contraindications and precautions, and dose adjustment criteria. Second, core relationships among these entities were extracted to support constrained reasoning; relation types included “recommended_for,” “contraindicated_in,” “dose_adjustment_for,” “not_recommended_with,” “covers_pathogen,” and other clinical-supporting relations. Third, the extracted information was organized into head-relation-tail triples, with guideline paragraphs or clauses retained as source metadata to enable evidence traceability.

During inference, the system retrieved knowledge snippets or subgraphs relevant to antibiotic selection, contraindications, and hepatic or renal dose adjustment based on the patient context and key laboratory indicators. These retrieved results were then provided to the downstream reasoning module as supporting evidence.

### Clinician-Defined Rule Constraints

To improve the safety and clinical compliance of the recommendations, we incorporated clinician-defined rule constraints curated by physicians. These rules covered care setting and severity stratification, pathogen risk and coverage strategies, restrictions and contraindications for combination therapy, and key boundary conditions related to hepatic and renal function. The rules were injected into the structured prompt as explicit constraint context to guide antibiotic selection and dose recommendation. The representative rules are provided in Table S2 in Multimedia Appendix 1.

### Hybrid-Context Reasoning

We integrated three sources of information into a unified hybrid context: (1) patient context, including admission narratives and key laboratory indicators; (2) experience context, including the top 5 similar cases and their antibiotic regimens; and (3) constraint context, including clinician-defined rule constraints and guideline or knowledge graph evidence retrieved using LightRAG.

During inference, the hybrid context was organized into a structured prompt and submitted to LightRAG in hybrid mode. All model inference was conducted in Chinese. The comprehensive prompt template, including the detailed instructions for context integration and reasoning steps, is provided in Multimedia Appendix 1. Guided by key entities and cues in the patient context, the system retrieved local entity-level details and global relational summaries related to antibiotic selection, contraindications, and hepatic or renal dose adjustment. These retrieved results were then combined with the patient context and rule constraints for downstream reasoning. On the basis of this integrated context, the model generated antibiotic and dose recommendations through a stepwise reasoning process. The final JSON array was extracted from each model response and parsed using Python’s json.loads() function (Python Software Foundation). If parsing failed or the output was empty, generation was repeated up to 5 times. No manual correction was performed.

### Model Implementation and Evaluation Metrics

We implemented the proposed pipeline using DeepSeek-V3, GLM-4.6, and GPT-4o. Detailed model implementation settings are provided in Multimedia Appendix 1. To examine the contribution of each module, we evaluated four categories of experimental settings: (1) a *base model* without retrieval augmentation or clinician-defined rule constraints; (2) *single-module variants*, in which only one component was added; (3) *dual-module variants*, in which 2 components were combined; and (4) the *full pipeline*, which integrated similar-case vector retrieval, guideline-based knowledge graph retrieval, and clinician-defined rule constraints.

The framework was evaluated on both the internal test set and the external validation cohort. Two tasks were assessed: *antibiotic selection* and *joint antibiotic selection plus dosing recommendation*. For the antibiotic selection task, only the standardized medication name was considered during matching. For the antibiotic selection plus dosing task, medication items were matched after standardizing drug name, dose, dosing frequency, and route of administration. Dose values were unit normalized when possible, and a 5% numeric tolerance was allowed to avoid penalizing minor unit conversion or formatting differences. Performance was measured using Jaccard accuracy, precision, recall, and *F*_1_-score. All metrics were calculated at the patient level based on true-positive, false-positive, and false-negative medication items and then averaged across cases. We used patient-level bootstrap resampling with 10,000 iterations to calculate 95% CIs for the performance metrics. To further assess robustness and clinical applicability, we conducted subgroup analyses in high-risk populations, including older patients and patients with hepatic or renal impairment. The detailed subgroup classification criteria are provided in Table S3 in Multimedia Appendix 1. Cross-site generalizability was examined using the external validation cohort.

### Ethical Considerations

This multicenter retrospective study was approved by the institutional ethics committees of Beijing Tsinghua Changgung Hospital (approval 23694-4-01) and Beijing Friendship Hospital (approval 2023-P2-031-01). The requirement for additional informed consent was waived by the ethics committees because this study used retrospective, deidentified clinical data and involved no direct patient contact or intervention. All extracted records were deidentified before analysis, and direct identifiers such as names, medical record numbers, phone numbers, and addresses were removed. The analytic data were stored in a secure research environment accessible only to authorized study personnel. No participants received compensation because this was a retrospective secondary analysis. No identifiable patient information appears in the manuscript figures, tables, or supplementary materials.

## Results

### Patient Characteristics and Study Cohorts

A total of 331 hospitalized patients with pneumonia were included in this study divided into a development cohort (n=233, 70.4% from Beijing Tsinghua Changgung Hospital) and an external validation cohort (n=98, 29.6% from Beijing Friendship Hospital). Baseline demographic characteristics, hepatic or renal impairment, and antibiotic class distributions are shown in Table 1. Compared with the external validation cohort, the development cohort included older patients and a higher proportion of male patients and patients with hepatic or renal impairment. The internal test set was broadly comparable to the overall development cohort in demographic and clinical characteristics, particularly in age and the prevalence of hepatic or renal dysfunction, although some variation was observed in antibiotic class distributions. Quinolones were the most commonly used antibiotic class in the development cohort, internal test set, and external validation cohort.

**Table 1.** Baseline demographic and clinical characteristics of patients in the development set, internal test set, and external validation set^a^.

Variable	Development set (n=233)	Internal test set (n=70)	External validation set (n=98)
Demographic characteristics			
Gender (man), n/N (%)	148/233 (63.5)	38/70 (54.3)	39/98 (39.8)
Age (y), median (IQR)	68.00 (59.00-75.00)	67.50 (60.00-74.00)	62.50 (54.25-73.00)
Advanced age (≥65 y), n/N (%)	149/233 (63.9)	47/70 (67.1)	43/98 (43.9)
Medical history, n/N (%)			
Hepatic or renal dysfunction	90/233 (38.6)	27/70 (38.6)	22/98 (22.4)
Antibiotic categories, n/N (%)			
Quinolones	147/368 (39.9)	52/98 (53.1)	79/110 (71.8)
β-lactamase inhibitor combinations	101/368 (27.4)	22/98 (22.4)	8/110 (7.3)
Antifungals	34/368 (9.2)	1/98 (1)	2/110 (1.8)
Cephalosporins	26/368 (7.1)	2/98 (2)	9/110 (8.2)
Carbapenems	15/368 (4.1)	4/98 (4.1)	7/110 (6.4)
Antivirals	13/368 (3.5)	4/98 (4.1)	0 (0)
Aminoglycosides	8/368 (2.2)	7/98 (7.1)	0 (0)
Tetracyclines	8/368 (2.2)	4/98 (4.1)	0 (0)
Sulfonamides	5/368 (1.4)	0 (0)	0 (0)
Glycopeptides and polypeptides	4/368 (1.1)	0 (0)	2/110 (1.8)
Macrolides	3/368 (0.8)	0 (0)	3/110 (2.7)
Oxazolidinones	4/368 (1.1)	2/98 (2)	0 (0)

a Percentages for antibiotic categories were calculated as the number of occurrences of each category divided by the total antibiotic category occurrences in each cohort (development cohort: n=368; internal test set: n=98; external validation cohort: n=110), reflecting prescription and category occurrence–level statistics.

### Performance for Antibiotic Selection and Joint Antibiotic Selection Plus Dosing Recommendation

We compared different LLMs and integration strategies across 2 tasks: antibiotic selection and joint antibiotic selection plus dosing recommendation (Table 2). On the internal test set, the full pipeline with DeepSeek-V3 achieved the best performance among all evaluated LLM-based methods, with an *F*_1_-score of 0.8110 (95% CI 0.7371-0.8762) and a Jaccard accuracy of 0.7624 (95% CI 0.6810-0.8386) for antibiotic selection and an *F*_1_-score of 0.7538 (95% CI 0.6671-0.8329) and a Jaccard accuracy of 0.7076 (95% CI 0.6145-0.7938) for joint antibiotic selection plus dosing recommendation. The full pipeline with GLM-4.6 and GPT-4o also outperformed their corresponding base models but remained inferior to the DeepSeek-V3–based implementation.

**Table 2.** Performance comparison between our model and the base large language models on the internal test set.

Model	*F*_1_-score(95% CI)	Jaccard accuracy (95% CI)	Precision (95% CI)	Recall (95% CI)
Our model+DeepSeek-V3				
Medication	*0.8110 (0.7371-0.8762)* ^a^	*0.7624 (0.6810-0.8386)*	*0.8429 (0.7690-0.9095)*	0.8119 (0.7357-0.8833)
Medication+dosing	*0.7538 (0.6671-0.8329)*	*0.7076 (0.6145-0.7938)*	*0.7833 (0.6952-0.8643)*	0.7524 (0.6619-0.8334)
Our model+GLM-4.6				
Medication	0.7101 (0.6286-0.7872)	0.6467 (0.5602-0.7321)	0.7619 (0.6761-0.8429)	0.7024 (0.6167-0.7857)
Medication+dosing	0.6734 (0.5814-0.7619)	0.6217 (0.5264-0.7164)	0.7238 (0.6262-0.8167)	0.6667 (0.5714-0.7595)
Our model+GPT-4o				
Medication	0.7714 (0.7181-0.8243)	0.6790 (0.6143-0.7476)	0.7048 (0.6405-0.7714)	*0.9143 (0.8595-0.9619)*
Medication+dosing	0.7257 (0.6662-0.7843)	0.6279 (0.5579-0.7017)	0.6571 (0.5929-0.7238)	*0.8690 (0.8024-0.9286)*
DeepSeek-V3				
Medication	0.4913 (0.4046-0.5753)	0.4110 (0.3274-0.4952)	0.4774 (0.3893-0.5619)	0.5381 (0.4429-0.6333)
Medication+dosing	0.4075 (0.3220-0.4952)	0.3336 (0.2550-0.4167)	0.3964 (0.3119-0.4833)	0.4476 (0.3500-0.5476)
GLM-4.6				
Medication	0.3793 (0.2941-0.4644)	0.3081 (0.2286-0.3883)	0.3667 (0.2821-0.4524)	0.4238 (0.3262-0.5190)
Medication+dosing	0.3317 (0.2503-0.4174)	0.2652 (0.1926-0.3450)	0.3214 (0.2405-0.4071)	0.3714 (0.2786-0.4690)
GPT-4o				
Medication	0.2939 (0.2265-0.3641)	0.2129 (0.1595-0.2712)	0.2679 (0.2048-0.3346)	0.3500 (0.2643-0.4381)
Medication+dosing	0.2060 (0.1436-0.2735)	0.1469 (0.0997-0.2000)	0.1845 (0.1286-0.2440)	0.2500 (0.1714-0.3381)

a Italicized values indicate the highest values.

On the external validation cohort, the full pipeline with DeepSeek-V3 maintained a strong performance (Table 3), achieving an *F*_1_ score of 0.8605 (95% CI 0.7891-0.9252) and a Jaccard accuracy of 0.8571 (95% CI 0.7857-0.9184) for antibiotic selection and an *F*_1_-score of 0.8503 (95% CI 0.7789-0.9150) and a Jaccard accuracy of 0.8469 (95% CI 0.7755-0.9133) for joint antibiotic selection plus dosing recommendation. In contrast, the base DeepSeek-V3 model without retrieval augmentation or clinician-defined rule constraints showed marked performance degradation, with an *F*_1_ score of 0.4163 (95% CI 0.3289-0.5031) and a Jaccard accuracy of 0.3801 (95% CI 0.2951-0.4660) for antibiotic selection and an *F*_1_ score of 0.3129 (95% CI 0.2303-0.3952) and a Jaccard accuracy of 0.2832 (95% CI 0.2049-0.3622) for joint antibiotic selection plus dosing recommendation.

**Table 3.** Performance comparison between our model and the base DeepSeek-V3 model on the external validation set.

Model	*F*_1_-score (95% CI)	Jaccard accuracy (95% CI)	Precision (95% CI)	Recall (95% CI)
Our model+DeepSeek-V3				
Medication	*0.8605 (0.7891-0.9252)* ^a^	*0.8571 (0.7857-0.9184)*	*0.8673 (0.7959-0.9286)*	*0.8571 (0.7857-0.9184)*
Medication+dosing	*0.8503 (0.7789-0.9150)*	*0.8469 (0.7755-0.9133)*	*0.8571 (0.7857-0.9184)*	*0.8469 (0.7755-0.9133)*
DeepSeek-V3				
Medication	0.4163 (0.3289-0.5031)	0.3801 (0.2951-0.4660)	0.3878 (0.3027-0.4728)	0.4728 (0.3741-0.5697)
Medication+dosing	0.3129 (0.2303-0.3952)	0.2832 (0.2049-0.3622)	0.2874 (0.2092-0.3673)	0.3622 (0.2704-0.4541)

a Italicized values indicate the highest values.

### Ablation Study

To assess the contribution of individual modules, we performed an ablation study using the full pipeline as the reference configuration. The configurations of all ablation variants are summarized in Table 4, and the corresponding performance results are presented in Table 5. In the antibiotic selection task, the full model achieved an *F*_1_-score of 0.8110. Removing clinician-defined rule constraints reduced the *F*_1_-score to 0.7429, removing similar-case vector retrieval reduced it to 0.7156, and removing guideline-based knowledge graph retrieval reduced it to 0.7843. A similar pattern was observed for joint antibiotic selection plus dosing recommendation, where the full model achieved an *F*_1_-score of 0.7538 compared with 0.6474 after removing rule constraints, 0.5946 after removing vector retrieval, and 0.7119 after removing guideline-based knowledge graph retrieval.

**Table 4.** Definitions of ablation configurations.

Ablation configuration	Similar-case vector retrieval	Guideline-based knowledge graph retrieval	Clinician-defined rule constraints
Our model	+^a^	+	+
Our model – GraphRAG^b^	+	–^c^	+
Our model – vector	–	+	+
Our model – rule	+	+	–
Our model – GraphRAG – vector	–	–	+
Our model – GraphRAG – rule	+	–	–
Our model – vector – rule	–	+	–
DeepSeek-V3	–	–	–

a Module included.

b GraphRAG: Graph Retrieval-Augmented Generation.

c Module removed.

**Table 5.** Ablation analysis of the contributions of individual modules to model performance.

Model	*F*_1_-score (95% CI)	Jaccard accuracy (95% CI)	Precision (95% CI)	Recall (95% CI)
Our model				
Medication	*0.8110 (0.7371-0.8762)* ^a^	*0.7624 (0.6810-0.8386)*	0.8429 (0.7690-0.9095)	*0.8119 (0.7357-0.8833)*
Medication+dosing	*0.7538 (0.6671-0.8329)*	*0.7076 (0.6145-0.7938)*	*0.7833 (0.6952-0.8643)*	*0.7524 (0.6619-0.8334)*
Our model – GraphRAG^b^				
Medication	0.7843 (0.7152-0.8476)	0.7217 (0.6417-0.7964)	*0.8452 (0.7738-0.9095)*	0.7738 (0.6976-0.8452)
Medication+dosing	0.7119 (0.6262-0.7929)	0.6538 (0.5633-0.7405)	0.7595 (0.6690-0.8429)	0.7071 (0.6167-0.7929)
Our model – rule				
Medication	0.7429 (0.6748-0.8077)	0.6633 (0.5857-0.7405)	0.7476 (0.6714-0.8214)	0.7952 (0.7190-0.8643)
Medication+dosing	0.6474 (0.5648-0.7262)	0.5683 (0.4836-0.6529)	0.6440 (0.5583-0.7286)	0.7000 (0.6095-0.7857)
Our model – vector				
Medication	0.7156 (0.6427-0.7857)	0.6381 (0.5595-0.7155)	0.8060 (0.7262-0.8821)	0.6905 (0.6095-0.7690)
Medication+dosing	0.5946 (0.5000-0.6850)	0.5310 (0.4381-0.6238)	0.6655 (0.5619-0.7643)	0.5714 (0.4762-0.6643)
Our model – GraphRAG – vector				
Medication	0.7233 (0.6548-0.7890)	0.6390 (0.5602-0.7176)	0.8095 (0.7357-0.8786)	0.6929 (0.6190-0.7667)
Medication+dosing	0.6233 (0.5362-0.7081)	0.5533 (0.4621-0.6445)	0.6810 (0.5857-0.7714)	0.6024 (0.5143-0.6881)
Our model – GraphRAG – rule				
Medication	0.6253 (0.5458-0.7012)	0.5367 (0.4545-0.6212)	0.5986 (0.5190-0.6786)	0.7143 (0.6262-0.7976)
Medication+dosing	0.5663 (0.4829-0.6486)	0.4843 (0.4000-0.5700)	0.5438 (0.4581-0.6310)	0.6452 (0.5500-0.7381)
Our model – vector – rule				
Medication	0.6447 (0.5589-0.7267)	0.5717 (0.4843-0.6581)	0.6298 (0.5405-0.7167)	0.7048 (0.6119-0.7976)
Medication+dosing	0.5641 (0.4770-0.6529)	0.4912 (0.4031-0.5814)	0.5417 (0.4536-0.6333)	0.6262 (0.5310-0.7214)
DeepSeek-V3				
Medication	0.4913 (0.4046-0.5753)	0.4110 (0.3274-0.4952)	0.4774 (0.3893-0.5619)	0.5381 (0.4429-0.6333)
Medication+dosing	0.4075 (0.3220-0.4952)	0.3336 (0.2550-0.4167)	0.3964 (0.3119-0.4833)	0.4476 (0.3500-0.5476)

a Italicized values indicate the highest values.

b GraphRAG: Graph Retrieval-Augmented Generation.

Performance declined further when multiple components were removed simultaneously. For example, the variant without guideline-based knowledge graph retrieval plus rule constraints achieved an *F*_1_-score of 0.6253 (95% CI 0.5458-0.7012) for antibiotic selection and 0.5663 (95% CI 0.4829-0.6486) for joint antibiotic selection plus dosing recommendation, whereas the variant without vector retrieval plus rule constraints achieved *F*_1_-scores of 0.6447 (95% CI 0.5589-0.7267) and 0.5641 (95% CI 0.4770-0.6529), respectively. The base model without retrieval augmentation or rule constraints showed the lowest overall performance.

### Subgroup Analyses by Hepatic or Renal Function and Age

**Figure 2.**
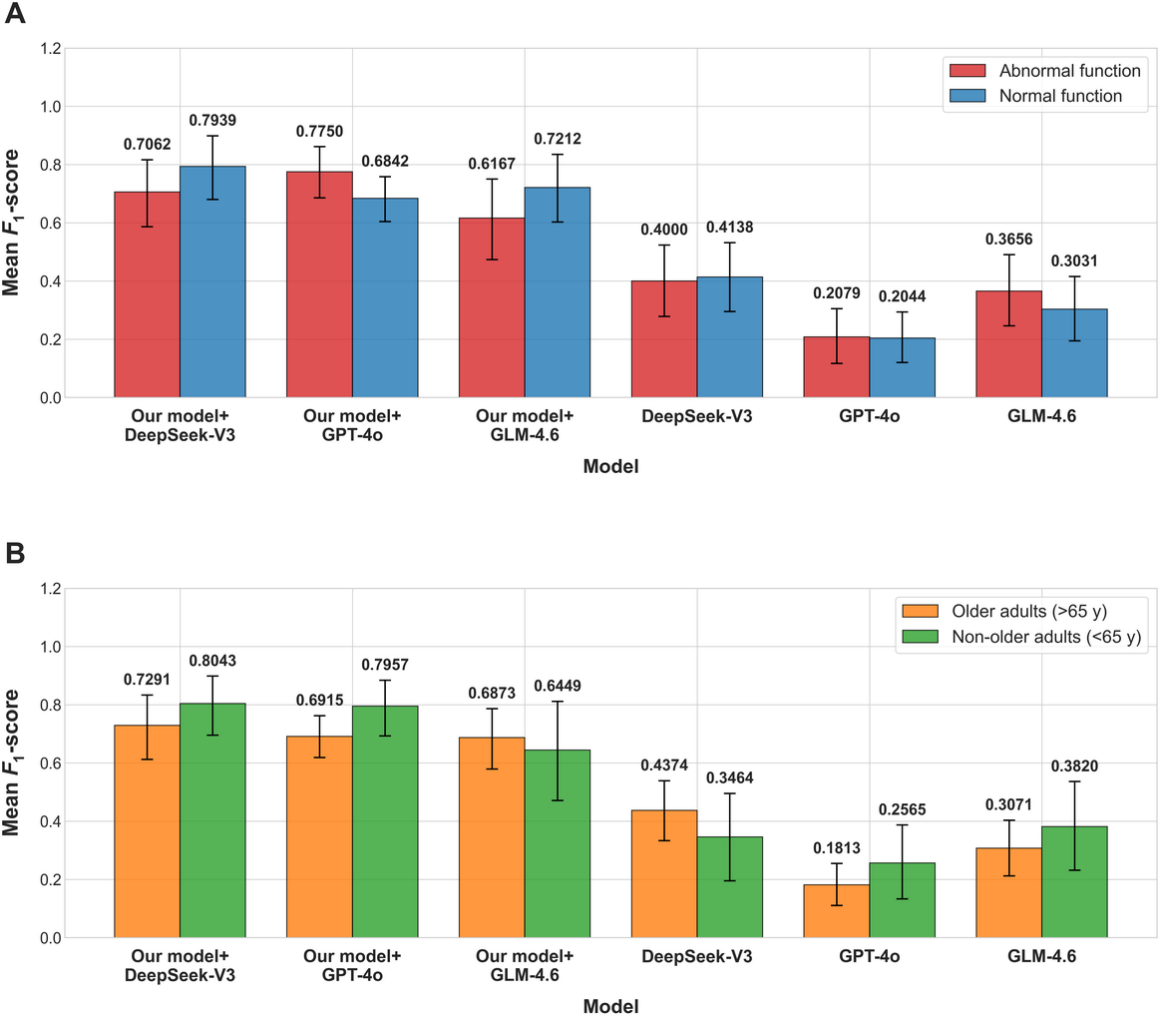
Subgroup analysis of model performance for the joint antibiotic selection plus dosing recommendation task on the internal test set by (A) hepatic and renal function and (B) age. Error bars represent 95% CIs.

We further evaluated model robustness for the joint antibiotic selection plus dosing recommendation task in clinically important subgroups defined by hepatic or renal function and age using the internal test set (n=70; Figure 2). In the hepatic or renal function subgroups, the full pipeline using DeepSeek-V3 achieved mean *F*_1_-scores of 0.7062 (95% CI 0.5823-0.8198) in patients with impairment and 0.7939 (95% CI 0.6798-0.8991) in those without impairment. In the age-based subgroups, the corresponding mean *F*_1_-scores were 0.7291 (95% CI 0.6177-0.8326) in patients aged 65 years or older and 0.8043 (95% CI 0.6884-0.9058) in those younger than 65 years. Overall, the integration strategy maintained relatively stable performance across all subgroups, whereas the base LLM approach consistently underperformed across the board.

### Error Analysis and Expert Evaluation

To further assess recommendation safety and reasoning quality, respiratory physicians reviewed 45 error cases and a random sample of 50 generated recommendations. Error cases were categorized as safe but suboptimal or potentially unsafe. Of the 45 reviewed error cases, 43 (95.6%) were classified as safe but suboptimal recommendations, whereas only 2 (4.4%) were considered potentially unsafe. Both unsafe cases were related to insufficient consideration of hepatic or renal function during antibiotic selection or dose recommendation.

For reasoning quality evaluation, physicians assessed 50 cases across 4 dimensions: evidence relevance, rule applicability, reasoning consistency, and hallucination risk. For evidence relevance, 88% (n=44) of the cases received the highest score, and 12% (n=6) of the cases received a partial relevance score, with no cases rated as irrelevant. All reviewed cases received acceptable ratings for rule applicability, reasoning consistency, and hallucination risk, with no cases judged to contain incorrect rule application, inconsistent reasoning, or clinically significant hallucinations.

## Discussion

### Principal Findings

In this multicenter retrospective study, we developed and validated a constrained LLM-based CDS pipeline for antibiotic selection and dose recommendation in hospitalized patients with pneumonia. The proposed framework, which integrates dual-branch retrieval, clinician-defined rule constraints, and hybrid-context reasoning, consistently outperformed the corresponding base LLMs across both the internal test set and the external validation cohort. It also maintained a relatively stable performance in clinically important high-risk subgroups, including older patients and patients with hepatic or renal impairment. Notably, the higher performance in the external validation cohort may be partly related to differences in patient characteristics and prescribing patterns between the 2 hospitals. The external validation cohort had a more concentrated antibiotic distribution and relatively simpler medication combinations, whereas the development cohort included a broader range of antibiotic categories and more complex regimens, which may have made the recommendation task more challenging. Together, these findings suggest that combining external evidence retrieval with explicit clinical constraints can improve the consistency and robustness and provide preliminary evidence of cross-site generalizability of LLM-assisted antimicrobial decision support.

The performance gains were attributable to the complementary contributions of multiple components rather than any single module. Ablation analyses showed consistent performance declines after removal of clinician-defined rule constraints, similar-case vector retrieval, or guideline-based knowledge graph retrieval, with larger reductions observed when multiple components were removed simultaneously (Table 5). These findings indicate that the 3 information sources served distinct but complementary functions: similar-case retrieval provided patient-aligned experience-based references; guideline-based knowledge graph retrieval contributed structured evidence-based knowledge; and clinician-defined rule constraints encoded explicit boundary conditions for dose adjustment, contraindications, and hepatic or renal function. In inpatient pneumonia management, where antibiotic decisions are both time sensitive and high risk, parametric LLM knowledge alone may be insufficient. By integrating patient-specific context, external evidence, and explicit clinical constraints within a unified reasoning framework, the proposed framework improved the consistency and safety of antibiotic recommendations. This design is also conceptually aligned with the early empirical decision-making process typically performed during infectious disease consultation while remaining intended as a clinician support tool rather than a replacement for specialist consultation or clinician judgment.

### Comparison to Prior Work

Compared with traditional black-box prediction models, the proposed framework emphasizes a more interpretable, traceable, and auditable form of CDS. The system is not intended to replace clinician judgment but to function as an assistive tool for antimicrobial stewardship and medication review in hospitalized patients with pneumonia. Its practical value lies in its ability to rapidly integrate admission narratives, key hepatic and renal function indicators, similar-case experience, and guideline-based evidence to generate candidate recommendations for antibiotic selection and dose recommendation. At the same time, the system provides traceable evidence and rule trigger information, which may facilitate rapid clinician verification and improve the transparency of the decision-making process. Given that the external validation cohort differed from the development cohort in age, sex distribution, hepatic or renal impairment, and antibiotic class distribution, whereas the full pipeline still maintained strong performance, the findings further support the potential applicability of this approach in heterogeneous real-world settings.

This study has several limitations. First, the sample size was relatively limited, and the data were derived from only 2 hospitals. In particular, the external validation cohort included only 98 patients, and the distribution of antibiotic categories was imbalanced. This limits the strength of conclusions regarding cross-site generalizability. Therefore, further validation in larger, multi-regional, multilevel, and more category-balanced health care settings is needed. Second, although the reference labels were adjudicated by experienced physicians, the source information included real-world antibiotic orders, which are influenced by clinician experience, local prescribing preferences, and institutional antimicrobial stewardship policies. Accordingly, these labels should not be interpreted as an absolute gold standard, and such label-level heterogeneity may affect model generalizability. In addition, the drug library in the prompt was sorted by common clinical use, which may have introduced positional or frequency-related bias; future evaluations should test alternative drug list orderings. Third, stable structured body weight and BMI information was not available in the retrospective dataset. Consequently, the framework could not fully evaluate individualized weight-based dosing strategies for antimicrobial agents that require body weight adjustment. Future studies should incorporate structured body weight information to support more precise dose recommendation. Fourth, antibiotic options, resistance patterns, and clinical guidelines continue to evolve over time, so the knowledge graph, rule base, and retrieval resources will require ongoing maintenance and updating. In addition, the guideline-based knowledge graph was constructed from publicly available guidelines; therefore, we cannot fully exclude the possibility that some guideline knowledge was already present in the evaluated LLMs’ parametric memory. Future work should further evaluate the framework using recently updated guidelines or institution-specific antimicrobial stewardship pathways to better isolate the contribution of retrieval augmentation. Fifth, there was a temporal mismatch between the development and external validation cohorts. Although patients with confirmed COVID-19 pneumonia were excluded, residual differences in pneumonia etiology and antimicrobial prescribing behavior across time may still affect cross-cohort comparability. Finally, this study was retrospective and did not include prospective evaluation in real clinical workflows. Future work should assess the framework in human-in-the-loop practice settings and further examine its effects on prescribing quality, antimicrobial stewardship, and clinical outcomes.

### Conclusions

In conclusion, we developed and externally validated a constrained LLM-based CDS pipeline for antibiotic selection and dose recommendation in hospitalized patients with pneumonia. By integrating dual-branch retrieval, clinician-defined rule constraints, and hybrid-context reasoning, the proposed framework improved the consistency and interpretability of LLM-assisted antimicrobial decision support and provided preliminary evidence of cross-site generalizability. The system also provided traceable evidence and rule trigger information to support clinician verification, highlighting its potential value for antimicrobial stewardship and medication review. Further prospective evaluation is needed to assess its performance and utility in real-world clinical workflows.

## Supplementary material

10.2196/98207 Multimedia Appendix 1 Prompt template, results, and technical implementation details.

## Abbreviations

CDS: clinical decision support
LLM: large language model
