# Supplementary material for: Large Language Model–Based Clinical Decision Support for Antibiotic Selection and Dose Recommendation in Hospitalized Patients With Pneumonia: Multicenter Retrospective Study
Source: JMIR Med Inform. 2026 Aug 4;14:e98207. doi: 10.2196/98207 (PMC13436791; doi:10.2196/98207)
Supplement: Supplementary file 1 [file medinform-v14-e98207-s001.docx]

**Appendix A: Prompt Templates**

**Appendix A1. Prompt Template for Final Antibiotic and Dose Recommendation**

**Chinese：**

```
你是一名经验丰富的呼吸内科医生，请根据病人的入院记录和化验结果，结合指南、规则与相似病例，精准预测最有可能的用药组合及剂量（仅抗感染药物）。
药物必须在药物库中选（按常用性排序）：
盐酸莫西沙星氯化钠注射液,左氧氟沙星氯化钠注射液,注射用头孢他啶,注射用哌拉西林钠他唑巴坦钠,注射用拉氧头孢钠,注射用头孢曲松钠,注射用头孢哌酮钠舒巴坦钠,注射用美罗培南,注射用氨曲南,注射用头孢唑肟钠,氟康唑氯化钠注射液,注射用亚胺培南西司他丁钠,注射用头孢美唑钠,注射用两性霉素B,伏立康唑分散片,注射用替加环素,奈玛特韦/利托那韦片,泊沙康唑口服混悬液,利奈唑胺葡萄糖注射液,注射用厄他培南,硫酸依替米星注射液,硫酸阿米卡星注射液,注射用阿奇霉素,磷酸奥司他韦胶囊,复方磺胺甲恶唑片,注射用阿莫西林钠克拉维酸钾,注射用盐酸万古霉素,艾沙康唑胶囊,注射用硫酸多粘菌素,注射用醋酸卡泊芬净,盐酸米诺环素胶囊,注射用替考拉宁,甲硝唑氯化钠注射液,阿兹夫定片,康替唑胺片,氟胞嘧啶片

### 1. 参考病例
{few_shot}

### 2. 核心规则
{rules}
### 3. 患者信息
病历：{record_text}
过敏史：{allergy}
化验：{lab_text}

### 4. 分析
基于知识图谱和经验分步思考：
1. 基于病史、影像描述、和化验指标等信息，判断最可能的肺部感染类型（区分细菌性/真菌性/病毒性），若入院记录中有确认病原体重点针对该病原体进行治疗，并评估肺炎严重程度（轻症、重症）。
2. 病人肝肾功能是否异常（分析化验指标）？病人是否有药物过敏？结合病人年龄、病情严重程度等信息进行分析。
3. 从知识图谱中检索相关知识，参考类似病例，并选取适合该病例的一些核心规则，汇总所有信息，确定最合理的药物组合和剂量，做到精准用药。
4. 先输出详细思考过程，然后在最后严格输出 JSON 数组格式的预测结果，包括药物名、具体剂量（多少g/mg Q12H/Q8H/BID...）、用药方式（IVF/PO...），例如：["左氧氟沙星氯化钠注射液（0.5g QD IVF）"，"硫酸依替米星注射液（200mg QD IVF）"，"伏立康唑片（100mg Q12H PO）"]。
```

**English：**

As an experienced respiratory physician, based on the patient's admission record and laboratory results, combined with guidelines, rules, and similar cases, accurately predict the most likely drug combination and dosage (anti-infective drugs only).

Drugs must be selected from the drug library (sorted by common usage):

Moxifloxacin Hydrochloride and Sodium Chloride Injection, Levofloxacin and Sodium Chloride Injection, Ceftazidime for Injection, Piperacillin Sodium and Tazobactam Sodium for Injection, Latamoxef Sodium for Injection, Ceftriaxone Sodium for Injection, Cefoperazone Sodium and Sulbactam Sodium for Injection, Meropenem for Injection, Aztreonam for Injection, Ceftizoxime Sodium for Injection, Fluconazole and Sodium Chloride Injection, Imipenem and Cilastatin Sodium for Injection, Cefmetazole Sodium for Injection, Amphotericin B for Injection, Voriconazole Dispersible Tablets, Tigecycline for Injection, Nirmatrelvir/Ritonavir Tablets, Posaconazole Oral Suspension, Linezolid and Glucose Injection, Ertapenem for Injection, Etimicin Sulfate Injection, Amikacin Sulfate Injection, Azithromycin for Injection, Oseltamivir Phosphate Capsules, Compound Sulfamethoxazole Tablets, Amoxicillin Sodium and Clavulanate Potassium for Injection, Vancomycin Hydrochloride for Injection, Isavuconazole Capsules, Colistin Sulfate for Injection, Caspofungin Acetate for Injection, Minocycline Hydrochloride Capsules, Teicoplanin for Injection, Metronidazole and Sodium Chloride Injection, Azvudine Tablets, Contezolid Tablets, Flucytosine Tablets

\### 1. Reference Cases

{few_shot}

\### 2. Core Rules

{rules}

\### 3. Patient Information

Medical Record: {record_text}

Allergy History: {allergy}

Laboratory Results: {lab_text}

\### 4. Analysis

Based on knowledge graph and experience, think step by step:

1. Based on medical history, imaging description, and laboratory indicators, determine the most likely type of pulmonary infection (differentiate bacterial/fungal/viral). If a pathogen is confirmed in the admission record, target treatment accordingly. Assess pneumonia severity (mild, severe).

2. Are there any abnormalities in liver and kidney function (analyze lab indicators)? Does the patient have any drug allergies? Analyze based on patient age, disease severity, and other factors.

3. Retrieve relevant knowledge from the knowledge graph, refer to similar cases, and select some core rules applicable to this case. Synthesize all information to determine the most reasonable drug combination and dosage for precise treatment.

4. First, output the detailed thinking process, then strictly output the prediction result in JSON array format, including drug name, specific dosage (how many g/mg Q12H/Q8H/BID...), and route of administration (IVF/PO...). For example: ["Levofloxacin and Sodium Chloride Injection (0.5g QD IVF)", "Etimicin Sulfate Injection (200mg QD IVF)", "Voriconazole Tablets (100mg Q12H PO)"].

**Appendix A2. Prompt Template for Similar-Case Summarization**

**Chinese:**

请将以下入院记录转化为【结构化的临床特征摘要】，用于匹配最相似的历史治疗方案。
【关键原则】：
1. **提炼**：年龄、基础疾病、感染症状、检查报告、病原学信息、肝肾功能(根据化验指标评估功能情况，分为四类：正常、轻度异常、中度异常、重度异常，仅输出结论，如肾功能轻度异常、肝功能正常)、既往史、过敏史、吸烟饮酒史、生命体征等重要信息。去除：无关、繁复的信息。
2. **数值定性化**：不要只写数字，要判断高低。例如 "CRP 98mg/L" -> "CRP显著升高"；"eGFR 45" -> "肾功能中度受损"。
3. **只留阳性**：不要包含"否认"、"无"的信息。如果无过敏，写"无过敏史"；如果无高血压，直接不写高血压。
4. **药物标准化**：将药品的商品名统一转为药品名。
5. 长度：300字以内，一段文字。

入院记录：{str(text)}
化验指标：{lab_text}

【输出示例】：
基本情况：老年女性，间断发热伴咳痰5月，伴咽痛、干咳、肌肉酸痛、乏力，诊断为肺炎和呼吸衰竭，需呼吸机辅助通气，体温38.5°C，血氧饱和度偏低。
检查和检验报告：胸部CT示左肺下叶炎症，血常规CRP显著升高，淋巴细胞减少。肝功能轻度异常，肾功能正常。
既往史：健康，无慢性基础病。无过敏史。
用药史：院外曾用莫西沙星治疗效果不佳。

**English:**

Please convert the following admission record into a structured clinical feature summary for matching the most similar historical treatment regimens.

Key principles:

1. Information extraction:

Extract important information, including age, underlying diseases, infection-related symptoms, examination findings, pathogen-related information, hepatic and renal function, past medical history, allergy history, smoking and drinking history, and vital signs. Hepatic and renal function should be assessed based on laboratory indicators and categorized into four levels: normal, mild abnormality, moderate abnormality, and severe abnormality. Only output the conclusion, such as “mild renal dysfunction” or “normal hepatic function.” Remove irrelevant and redundant information.

2. Qualitative interpretation of numerical values:

Do not only retain raw numerical values. Interpret their clinical meaning. For example:

“CRP 98 mg/L” should be converted to “markedly elevated CRP.”

“eGFR 45” should be converted to “moderate renal impairment.”

3. Retain only positive and key negative information:

Do not include large amounts of low-value negative information such as “denies” or “no.” If there is no allergy history, write “no known allergy history.” If the patient has no hypertension, it is not necessary to write “no hypertension.”

4. Medication standardization:

Convert brand names of medications into generic drug names.

5. Length:

Limit the summary to within 300 Chinese characters and output it as one paragraph.

Admission record:

{str(text)}

Laboratory indicators:

{lab_text}

Output example:

Basic information: Elderly female patient with intermittent fever and productive cough for 5 months, accompanied by sore throat, dry cough, myalgia, and fatigue. Diagnosed with pneumonia and respiratory failure, requiring ventilator-assisted ventilation. Body temperature was 38.5°C and oxygen saturation was low. Examinations and laboratory findings: Chest CT showed inflammation in the left lower lobe. CRP was markedly elevated and lymphocyte count was decreased. Mild hepatic dysfunction and normal renal function were observed. Past medical history: Generally healthy, without chronic underlying diseases. No known allergy history. Medication history: Prior outpatient treatment with moxifloxacin was ineffective.

**Appendix B：Supplementary Tables**

**Supplementary Table 1. Hepatic and Renal Function Indicators Used in the Study**

| **Category** | **Indicator Name** | **Abbreviation** | **Unit** |
| --- | --- | --- | --- |
| Hepatic Function | Alanine Aminotransferase | ALT | U/L |
|  | Aspartate Aminotransferase | AST | U/L |
|  | Total Bilirubin | TBIL | μmol/L |
|  | Direct Bilirubin | DBIL | μmol/L |
| Renal Function | Creatinine | Cre | μmol/L |
|  | Estimated Glomerular Filtration Rate | eGFR | mL/min/1.73m^2^ |
|  | Blood Urea Nitrogen | BUN | mmol/L |

**Supplementary Table 2. Representative Clinical Rules in Chinese and English**

| Category | Clinical Rules (Chinese) | Clinical Rules (English) |
| --- | --- | --- |
| Core Decision Principles | 优先选择一天一次（QD）的药物（如左氧、头孢曲松）以提高依从性。 | Prefer Once-Daily (QD) agents (e.g., Levofloxacin, Ceftriaxone) to enhance adherence. |
| Site-Specific Regimens | ICU 重症：必须静脉给药，首选广谱方案联合喹诺酮类或大环内酯类。 | ICU (Severe): Must use IV administration; initial choice includes broad-spectrum combinations. |
| Pathogen-Specific Strategies | 非典型病原体（如支原体）：首选呼吸喹诺酮类（莫西/左氧）实现双覆盖。 | Atypical Pathogens: Preferred respiratory quinolone monotherapy for dual coverage. |
| Key Drug Selection & Pitfalls | 头孢曲松使用逻辑：适合细菌感染患者，若需覆盖支原体必须联合大环内酯类。 | Ceftriaxone Logic: For confirmed bacterial infection; must combine with macrolides for Mycoplasma. |
| Combination Contraindications | 严禁盲目联用：左氧/莫西原则上不与哌拉西林/他唑巴坦联用。 | Avoid Unjustified Combinations: Avoid combining quinolones with Piperacillin/Tazobactam. |

**Supplementary Table 3. Criteria for subgroup classification**

| **Variable** | **Threshold** | **Subgroup definition** |
| --- | --- | --- |
| BUN | >7.5 mmol/L | Abnormal hepatic or renal function if any one abnormal threshold met |
| eGFR | <60 mL/min/1.73 m² |  |
| Cre | >109 μmol/L (Male) or >79 μmol/L (Female) |  |
| ALT | >40 U/L |  |
| AST | >40 U/L |  |
| TBIL | >21 μmol/L |  |
| DBIL | >8 μmol/L |  |
| Age | ≥65 years | Elderly subgroup |

**Supplementary Table 4. Criteria for expert evaluation of reasoning quality and evidence fidelity**

| **Dimension** | **Score** | **Definition** |
| --- | --- | --- |
| Evidence relevance | 2 | Retrieved evidence is highly relevant to the current case and supports antibiotic selection or dose recommendation. |
|  | 1 | Retrieved evidence is partially relevant and supports only part of the decision-making process. |
|  | 0 | Retrieved evidence is weakly related or not useful for the current case. |
| Rule applicability | 2 | Applied rules appropriately match the clinical conditions of the current case. |
|  | 1 | Rules are partially applicable because of incomplete information or partial condition matching. |
|  | 0 | Rules are clearly inapplicable or incorrectly applied. |
| Reasoning faithfulness | 2 | Reasoning is supported by case information, laboratory findings, retrieved evidence, or rules without unsupported inferences. |
|  | 1 | Reasoning is generally reasonable but contains minor extrapolation, omission, or insufficient justification. |
|  | 0 | Reasoning contradicts case facts or retrieved evidence, or relies on nonexistent information. |
| Hallucination | 2 | No obvious unsupported or fabricated information identified. |
|  | 1 | Minor unsupported statements are present but do not materially affect the recommendation. |
|  | 0 | Clinically important fabricated information is present and may affect treatment decisions or safety assessment. |

**Appendix C:** **Technical Implementation Details**

### 1. LLM generation settings

| **Model** | **API name** | **API version** | **Temperature** |
| --- | --- | --- | --- |
| DeepSeek-v3 | deepseek-chat | 2025-08-21 | 1.0 (default) |
| GPT-4o | gpt-4o | 2024-11-20 | 1.0 (default) |
| GLM-4.6 | glm-4.6 | 2025-09-30 | 0.7 (default) |

### 2. Embedding and similar-case retrieval settings

| **Setting** | **Value** |
| --- | --- |
| Embedding model | bge-m3:latest |
| Embedding dimension | 1024 |
| Max embedding tokens | 8192 |
| Similarity metric | cosine similarity |
| Case summary language | Chinese |

### 3. LightRAG and retrieval settings

| **Setting** | **Value** |
| --- | --- |
| Version | LightRAG-1.4.4 |
| Retrieval mode | Hybrid |
| Embedding function | Ollama embedding |
| Embedding model | bge-m3:latest |
